# Impact of molecular surgical margin analysis on the prediction of pancreatic cancer recurrences after pancreaticoduodenectomy

**DOI:** 10.1186/s13148-021-01165-8

**Published:** 2021-09-16

**Authors:** Yuki Sunagawa, Masamichi Hayashi, Suguru Yamada, Hiroshi Tanabe, Keisuke Kurimoto, Nobutake Tanaka, Fuminori Sonohara, Yoshikuni Inokawa, Hideki Takami, Mitsuro Kanda, Chie Tanaka, Goro Nakayama, Masahiko Koike, Yasuhiro Kodera

**Affiliations:** Department of Gastroenterological Surgery (Surgery II), Nagoya University Graduate School of Medicine, 65 Tsurumai-cho, Showa-ku, Nagoya, Aichi 466-8560 Japan

**Keywords:** Pancreatic cancer, Surgical margin, Methylation

## Abstract

**Background:**

Pancreatic cancer is one of the lethal cancers among solid malignancies. Pathological diagnosis of surgical margins is sometimes unreliable due to tissue shrinkage, invisible field cancerization and skipped lesions like tumor budding. As a result, tumor recurrences sometimes occur even from the pathologically negative surgical margins.

**Methods:**

We applied molecular surgical margin (MSM) analysis by tissue imprinting procedure to improve the detection sensitivity of tiny cancerous cells on the surgical specimen surface after pancreatoduodenectomy. Surgical specimens were collected from 45 pancreatic cancer cases who received subtotal stomach preserving pancreatoduodenectomy at Nagoya University Hospital during 2017–2019. Quantitative methylation-specific PCR (QMSP) of the original methylation marker panel (*CD1D*, *KCNK12*, *PAX5*) were performed and analyzed with postoperative survival outcomes.

**Results:**

Among 45 tumors, 26 cases (58%) were QMSP-positive for *CD1D*, 25 (56%) for *KCNK12* and 27 (60%) for *PAX5*. Among the 38 tumors in which at least one of the three markers was positive, *CD1D*-positive cancer cells, *KCNK12*-positive cancer cells, and *PAX5*-positive cancer cells were detected at the surgical margin in 8 cases, 7 cases and 10 cases, respectively. Consequently, a total of 17 patients had at least one marker detected at the surgical margin by QMSP, and these patients were defined as MSM-positive. They were associated with significantly poor recurrence-free survival (*p* = 0.002) and overall survival (*p* = 0.005) than MSM-negative patients. Multivariable analysis showed that MSM-positive was the only significant independent factor for worse recurrence-free survival (hazard ratio: 3.522, 95% confidence interval: 1.352–9.179, *p* = 0.010). On the other hand, a significant proportion of MSM-negative cases were found to have received neoadjuvant chemotherapy (*p* = 0.019).

**Conclusion:**

Pancreatic cancer-specific methylation marker panel was established to perform MSM analysis. MSM-positive status might represent microscopically undetectable cancer cells on the surgical margin and might influence the postoperative long-term outcomes.

**Supplementary Information:**

The online version contains supplementary material available at 10.1186/s13148-021-01165-8.

## Background

Pancreatic cancer (PC) is one of the most aggressive malignancies, the seventh leading cause of cancer-associated mortality in 2018 [1]. Since the incidence of PC increases, PC is estimated to be the second leading cause of cancer-associated deaths in the United States by 2030 [2]. Although surgical resection is the only potentially curative treatment for PC, only 20% of newly diagnosed patients have an indication for surgical resection [3]. Moreover, the 5-year survival rate after surgery does not exceed 20–25% [4], although surgical techniques and perioperative chemotherapy have been improving [5–7].

We previously reported that dissected peripancreatic tissue margin (DPM) negative PC resections could reduce the recurrence rate and improve the prognosis [8]. However, we sometimes experience local recurrences derived from the histologically DPM negative facets. It may partially because the histological diagnosis of surgical margins is usually tricky due to tissue shrinkage, skipped lesions like tumor budding [9, 10]. Also, the concept of 'field cancerization' implies that cancer-specific gene alterations possibly occur and spread through the pancreatic duct surrounding the primary tumor-originated pancreatic duct [11].

Molecular surgical margin analysis (MSM) by tissue imprinting procedure [12] has been reported as a super sensitive and quick method for evaluating the surgical margins of operative specimens [13, 14]. After the pancreatoduodenectomy for a pancreatic head cancer case, the resected specimen usually has a sizeable surgical margin area, including the forward serosa, backward connective tissue, and portal vein notch. In these cases, tissue imprinting procedure by nitrocellulose membranes [12] is suitable to collect tiny cells on the complicated surgical margin surfaces, compared with surgical margin tissue collection [13].

We used a PC-specific methylation marker panel instead of KRAS mutation to perform a molecular-based quick and super-sensitive diagnosis of the tiny cancerous cell existence because of their high sensitivity and rapidity. Kisiel JB et al. reported several methylation markers for PC by bisulfite DNA sequencing [15]. The top two sensitive markers that can distinguish PC from normal pancreas rather than KRAS mutation and one promising candidate marker from our previous study [16] were chosen for this study. We optimized quantitative methylation-specific PCR (QMSP) assay for them.

In this study, we tried to apply the tissue imprinting procedure to resected PC specimens after pancreatoduodenectomy. We then assessed whether PC-specific methylation detection on the surgical margin could predict cancer recurrences and patients' survival outcomes after surgery more precisely rather than several factors, including preoperative serum tumor markers, histological findings and tumor stages.

## Results

### QMSP values between tumor and adjacent normal pancreas

**Fig. 1:**
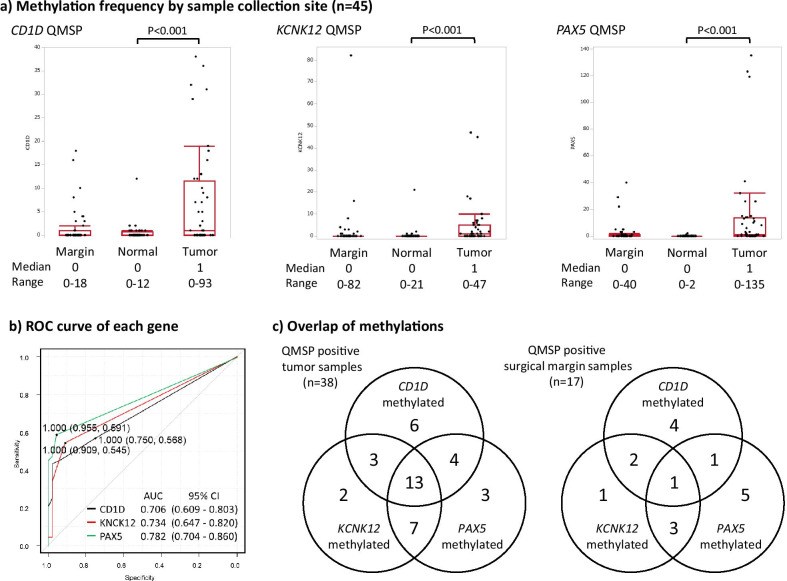
**a** Three gene methylation frequencies of surgical margin imprinting samples, adjacent normal pancreas and cancer tissues were examined and compared. **b** ROC curves of each gene of the panel to discriminate tumor from normal were shown. **c** Among 45 tested cases, any of three methylation marker positivity was detected in 38 cases. The overlapped distribution was shown in the left figure. Among 38 cases, 17 cases were molecular surgical margin positive. The overlapped distribution was shown in the right figure

QMSP values of all 45 samples were shown in Fig. 1a. In each gene of the panel, Tumor QMSP was significantly higher than normal QMSP (*CD1D*: *p* < 0.001, *KCNK12*: *p* < 0.001, *PAX5*: *p* < 0.001). It means that each candidate gene was significantly methylated in PCs rather than the adjacent normal pancreas. ROC curves of each gene to discriminate tumor from normal were shown in Fig. 1b. Interestingly, we realized that a certain number of surgical margin samples have relatively high QMSP values. Representative assays of molecular surgical margin analysis were shown in Additional file 1: Figure S1. All of the margin samples had an amplification curve on the *ACTB* assay as a reference gene. While, some of them had the curve on some methylation signals, defined as MSM positive (a). Others had non of methylation signals, which was defined as MSM negative (b).

### Diagnosis of molecular surgical margin analysis

For each gene of the methylation panel, MSM analysis positive (MSM-positive) was defined as the case with both tumor and surgical margin QMSP-positive using the optimal cut-off value. The rest of all cases were MSM-negative. As for *CD1D*, QMSP-positive tumors and adjacent normal tissues were 26 cases (58%) and 11 cases (24%) of 45 cases. Among them, QMSP-positive surgical margins were 8 cases and QMSP-negative in 18 cases. For *KCNK12*, QMSP-positive tumors and adjacent normal tissues were 25 cases (56%) and 4 cases (9%) of 45 cases, and QMSP-positive surgical margins were 7 cases and QMSP-negative in 18 cases. For *PAX5*, QMSP-positive tumors and adjacent normal tissues were 27 cases (60%) and 4 cases (9%) of 45 cases, and QMSP-positive surgical margins were 10 cases and QMSP-negative in 17 cases.

We summarized the QMSP results in Fig. 1c. Among 45 tumors, at least one gene methylation was found in 38 tumors (sensitivity 84.4% [95%CI 70.5–93.5], specificity 82.2 [95%CI 67.9–92.0], positive predictive value 82.6 [95%CI 68.6–92.2], negative predictive value 84.1 [95%CI 69.9–93.4]). It means that 84% of PC samples can be diagnosed by our methylation marker panel, while the rest of the 7 cases has to be excluded in this study and needs other tumor-specific gene markers. Then, among 38 cases, QMSP-positive surgical margin samples were found in 17 cases diagnosed as MSM-positive. Clinicohistological characteristics of these 17 cases were compared with the rest of 21 MSM analysis negative (MSM-negative) cases in Table 1. Interestingly, cases that underwent neoadjuvant chemotherapy had a significantly low risk of MSM-positive rather than no neoadjuvant chemotherapy cases (*p* = 0.019).

**Table 1:** Clinicopathological features stratified by QMSP

Valiables		All (*n* = 38)	Molecular surgical margin analysis		
			Negative (*n* = 21)	Positive (*n* = 17)	*p* value
Age, years	Median (range)	66 (36–86)	64 (36–86	72 (49–82)	0.735
Sex, *n* (%)	Female	13 (34.2)	7 (33.3)	6 (35.3)	1
	Male	25 (65.8)	14 (66.7)	11 (64.7)	
Tumor size, cm	Median (range)	2.3 (0.9–4.3)	2.5 (0.9–3.5)	2.1 (1.0–4.3)	0.401
Initial resectability, *n* (%)	Resectable	16	6	10	0.133
	Borderline resectable	15	11	4	
	Unresectable	7	4	3	
NAT, *n* (%)	Yes	24 (63.2)	17 (81.0)	7 (41.2)	**0.019**
	No	14 (36.8)	4 (19.0)	10 (58.8)	
Radiation, *n* (%)	Yes	3 (7.9)	3 (14.3)	0 (0)	0.238
	No	35 (92.1)	18 (85.7)	17 (100)	
CA19-9, U/ml	Median (range)	51.5 (1–5790	51 (1–5790	53 (1–1020)	0.871
DUPAN-2, U/ml	Median (range)	61.5 (25–1000)	47 (25–590)	120 (25–1000)	0.121
Pathological surgical margin, *n* (%)	Positive	11 (28.9)	6 (28.6)	5 (29.4)	1
	Negative	27 (71.1)	15 (71.4)	12 (70.6)	
Pathological surgical margin, μm	Median (range)	829 (0–4000)	1081 (0–4000)	372 (0–2648)	0.521
CY, *n* (%)	Positive	3 (7.9)	1 (4.8)	2 (11.8)	0.577
	Negative	35 (92.1)	20 (95.2)	15 (88.2)	
n, *n* (%)	Positive	6 (15.8)	5 (23.8)	1 (5.9)	0.197
	Negative	32 (84.2)	16 (76.2)	16 (94.1)	
ly, *n* (%)	Positive	23 (60.5)	12 (57.1)	11 (64.7)	0.744
	Negative	15 (39.5)	9 (42.9)	6 (35.3)	
v, *n* (%)	Positive	16 (42.1)	7 (33.3)	9 (52.9)	0.324
	Negative	22 (57.9)	14 (66.7)	8 (47.1)	
s, *n* (%)	Positive	29 (76.3)	16 (76.2)	13 (76.5)	1
	Negative	9 (23.7)	5 (23.8)	4 (23.5)	
rp, *n* (%)	Positive	29 (76.3)	15 (71.4)	14 (82.4)	0.476
	Negative	9 (23.7)	6 (28.6)	3 (17.6)	
ch, *n* (%)	Positive	12 (31.6)	6 (28.6)	6 (25.3)	0.734
	Negative	26 (68.4)	15 (71.4)	11 (64.7)	
du, *n* (%)	Positive	15 (39.5)	7 (33.3)	8 (47.1)	0.509
	Negative	23 (60.5)	14 (66.7)	9 (52.9)	
pv, *n* (%)	Positive	12 (31.6)	7 (33.3)	5 (29.4)	1
	Negative	26 (68.4)	14 (66.7)	12 (70.6)	

Bold indicates *P* < 0.05

NAT; neoadjuvant chemotherapy, CY; Peritoneal washing cytology, n; Lymph node metastasis, ly; Lymphatic invasion, v; Venous invasion, s; Serosal side of the anterior pancreatic invasion, rp; Retropancreatic tissue invasion, ch; Bile duct invasion, du; Duodenal invasion, pv; Portal vein system invasion

### Correlation between MSM analysis and DPM

**Fig. 2:**
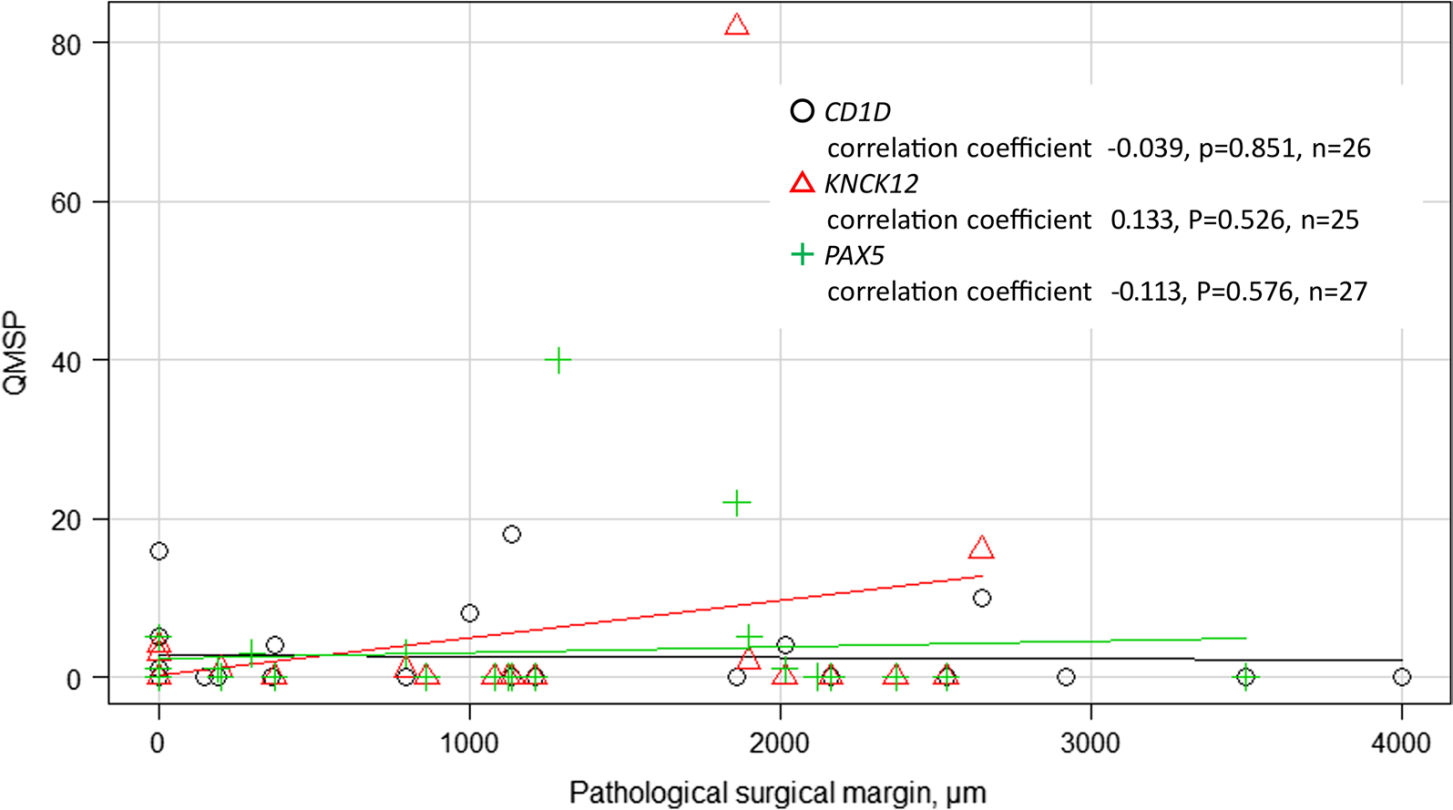
Correlations between pathological surgical margin distances with QMSP values of three genes were shown. No apparent correlation was found in any of the three gene methylation frequencies

We just compared MSM positivity with pathological positivity for all 38 samples. The number of pathological surgical margin (PSM) positive cases was 6 (29%) in MSM-negative cases and 5 (29%) in MSM-positive cases, with no significant difference (*p* = 0.999). Moreover, the median PSM distance was 1081 µm (range 0–4000) in MSM-negative cases and 372 µm (range 0–2648) in MSM-positive cases, with no significant difference (*p* = 0.521) (Table 1). There was no correlation between each QMSP value on surgical margin and PSM distance (*p* = 0.697 in *CD1D*, *p* = 0.557 in *KCNK12*, *p* = 0.576 in *PAX5*, Fig. 2).

### Postoperative prognostic outcomes of MSM positive cases

**Fig. 3:**
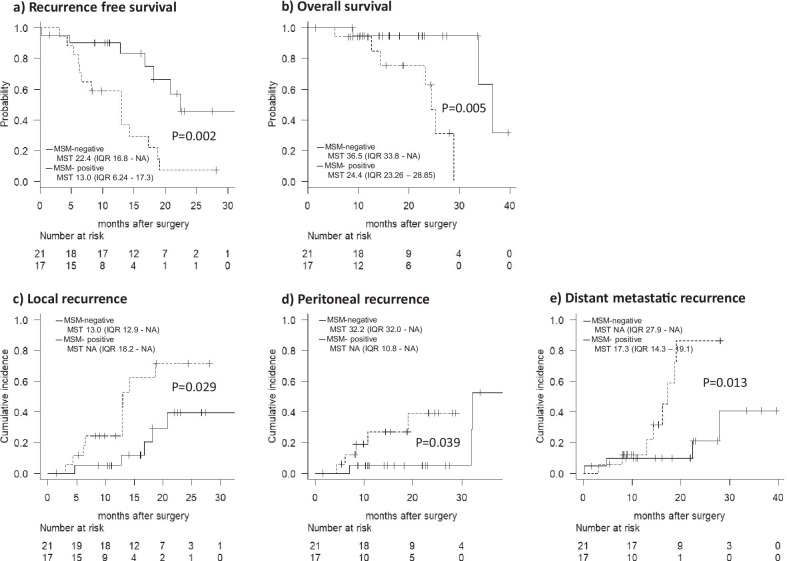
**a** Recurrence-free survival rate of QMSP positive cases and negative cases were compared. **b** Overall survival rate of QMSP positive cases and negative cases were compared. **c**–**e** Recurrence rate of QMSP positive cases and negative cases were compared depending on recurrence patterns, including local recurrences (**c**), peritoneal recurrences (**d**) and distant organ recurrences (**e**)

MSM-positive 17 cases were compared with MSM-negative 21 cases in postoperative prognostic outcomes (Fig. 3). There were significant differences in Recurrence-free survival (RFS) and Overall survival (OS) between the MSM-negative and MSM-positive groups (median RFS: 22.4 vs. 13.0 months, *p* = 0.002; median OS: 36.5 vs. 24.4 months, *p* = 0.005, Fig. 3a, b). Considering by recurrence type, all of the cumulative local recurrence (median local RFS: not reached vs. 13.0 months, *p* = 0.029, Fig. 3c), peritoneal recurrence (median peritoneal RFS: 32.2 vs. not reached months, *p* = 0.039, Fig. 3d) and distant metastatic recurrence (median distant metastatic RFS: not reached vs. 17.3 months, *p* = 0.013, Fig. 3e) were easy to occur in QMSP positive cases.

Even among PSM negative cases, significant differences were observed again in RFS and OS between the MSM-negative and MSM-positive groups (median RFS: 22.4 vs. 10.6 months, *p* = 0.016; median OS: 33.8 vs. 24.4 months, *p* = 0.043, Additional file 1: Figure S2a, S2b). Considering by recurrence type, MSM-positive cases had a tendency of cumulative local recurrence (median local RFS: not reached vs. 14.3 months, *p* = 0.072, Additional file 1: Figure S2c) and peritoneal recurrence (median peritoneal RFS not reached vs. not reached, *p* = 0.071, Additional file 1: Figure S2d). Whereas there was no significant difference in the distant metastatic recurrence (median distant metastatic RFS: 27.9 vs. 14.3 months, *p* = 0.117, Additional file 1: Figure S2e).

### Univariate and multivariate analyses of prognosis

In total 38samples, univariate analysis revealed that the MSM-positive and venous invasion factors were significant predictors of worse RFS. When the multivariable analysis was performed on these predictors, MSM-positive was the only significant independent factor for worse RFS (hazard ratio: 3.522, 95% confidence interval: 1.352–9.179, *p* = 0.010, Table 2).

**Table 2:** Univariate and multivariate cox proportional hazards regression analysis for recurrence free survival

	RFS					
	Univariate analysis			Multivariate analysis		
	HR	95% CI	*p* value	HR	95% CI	*p* value
Age (≥ 66 vs. < 66)	1.489	0.620–3.576	0.373			
Gender (Male vs. female)	0.559	0.235–1.331	0.189			
Tumor size at diagnosis (> 2 cm vs. ≤ 2 cm)	0.835	0.322–2.170	0.712			
NAT (yes vs. no)	0.952	0.400–2.269	0.912			
Radiation (yes vs no)	0.365	0.048–2.755	0.329			
CA 19-9 (> 37 U/ml vs. ≤ 37 U/ml)	1.780	0.717–4.417	0.214			
DUPAN-2 (> 150 U/ml vs. ≤ 150 U/ml)	2.201	0.931–5.204	0.073			
Pathological surgical margin (positive vs. negative)	0.762	0.295–1.969	0.575			
Pathological surgical margin (≤ 1 mm vs. > 1 mm)	0.686	0.287–1.641	0.397			
Molecular surgical margin (positive vs. negative)	3.989	1.569–10.14	**0.004**	3.522	1.352–9.179	**0.010**
CY (positive vs. negative)	0.767	0.177–3.320	0.722			
n (positive vs. negative)	0.275	0.037–2.048	0.207			
ly (positive vs. negative)	2.706	0.986–7.425	0.053			
v (positive vs. negative)	2.841	1.178–6.848	**0.020**	2.323	0.936–5.769	0.069
s (positive vs. negative)	1.672	0.558–5.012	0.359			
rp (positive vs. negative)	1.876	0.630–5.590	0.259			
ch (positive vs. negative)	1.735	0.718–4.196	0.221			
du (positive vs. negative)	1.249	0.525–2.972	0.615			
pv (positive vs. negative)	0.876	0.351–2.188	0.777			

Bold indicates *P* < 0.05

NAT; neoadjuvant chemotherapy, CY; Peritoneal washing cytology, n; Lymph node metastasis, ly; Lymphatic invasion, v; Venous invasion, s; Serosal side of the anterior pancreatic invasion, rp; Retropancreatic tissue invasion, ch; Bile duct invasion, du; Duodenal invasion, pv; Portal vein system invasion

## Discussion

MSM-positive cases were significantly associated with poor survival outcomes. Surprisingly, even in the PSM negative cases, MSM-positive cases exist and are also associated with poor survival outcomes. These results may imply that the judgment of PSM is sometimes tricky because of postoperative deformation of the modification by tissue shrinkage, neoadjuvant chemotherapy, tumor budding [9, 10], misunderstanding of actual surgical margin and limited slice of the tumor margin area. On the other hand, MSM analysis can collect cells from a whole area of the dissected surface, and the results were judged digitally. Thus it has fewer false negatives than pathological diagnosis if the ideal molecular marker panel is available [12]. However, it is necessary to consider that MSM analysis may count cancer cell contamination floating on the specimen's surface, which causes false positives due to its high sensitivity.

Interestingly, MSM-negative cases had significantly many neoadjuvant therapy (NAT) treated cases. Initial treatment before resection may effectively cause a tumor shrinkage and achieve not only PSM-negative but also MSM-negative. Regimens of 24 NAT includes 15 gemcitabine-base chemotherapy, six oxaliplatin-based chemotherapy and three S-1-based chemoradiotherapy. Although we cannot distinguish differences depending on each regimen in this small number study, it seems evident that NAT is effective for pancreatic cancer cases for keeping the molecularly safe tumor margins.

PCs often recurs postoperatively due to locally progressive intensity and anatomically resectable limits. Interestingly, postoperative recurrence is present in patients with pathologically positive margins and pathologically negative margins [8, 17, 18]. There are several MSM-positive cases among PSM negative cases in this study, and occult local cancer remnants may be involved in postoperative recurrences. Besides, MSM-positive cases also correlate distant organ metastases and peritoneal recurrences. Conversely, performing the local dissection with molecularly safe surgical margins is fundamental to avoid or postpone any type of postoperative recurrences.

The whole MSM analysis procedure takes less than 3 h from sampling to acquiring the QMSP result if methylation marker panel was available [13, 14]. Further improvement of the DNA extraction kit or real-time PCR procedure might minimize the time for utilizing it as an intraoperative application. If the result of MSM analysis is positive during surgery, additional resection or additional postoperative local therapy can be considered. To improve accuracy, imprinting the residual organ's surface after resection might be suitable rather than imprinting the resected specimen's surface to detect the occult remnant cells with epigenetic abnormalities.

There are a few limitations in this study. First, this study is a single-center analysis with a small number of samples. The positive rate of any methylation marker in tumors was 38 of 45 (84%). Thus 7 cases were excluded from the study. Although it may partially due to the preoperative chemotherapy, it is better to examine another cancer-specific methylation markers derived from some promising papers [19, 20] in the future study. Secondly, since MSM analysis is imprinted in manually, there is a possibility of variation in imprinting. However, in this study, sample collection was performed by a single experienced person [13, 14]. For clinical application, it is necessary to establish a protocol of imprinting. Finally, this cohort mainly consists of resectable PC without neoadjuvant chemotherapy and borderline resectable or unresectable PC with intensive neoadjuvant chemotherapy just because the routine neoadjuvant therapy still has not been established for resectable PC at that time. This situation may affect univariate analyses of RFS results.

## Conclusion

A pancreatic cancer-specific methylation marker panel was established and enabled us to perform MSM analysis. MSM-positive margin is one of the predictors of recurrence and survival in patients who underwent pancreaticoduodenectomy for PC. Additionally, performing NAT has the advantage of securing molecular negative surgical margins.

## Methods

### Patient cohort

Forty-eight PC cases who underwent curative-intent subtotal stomach-preserving pancreaticoduodenectomy at the Department of Gastroenterological Surgery (Surgery II), Nagoya University Graduate School of Medicine, Nagoya, Japan, between January 2017 and December 2019 were included. Of those, excluding 3 cases that were histologically proven to be non-invasive PCs, 45 cases were eligible for the study. Tumors, adjacent normal tissues and surgical margin imprinting samples were collected just after the surgical specimen was picked out from the surgical site. This study was approved by the hospital's ethics committee, and written informed consent was obtained from all patients about the use of surgical samples and their clinical data. The background and clinicopathological features of the patients are summarized in Table 3.

**Table 3:** Patient characteristics (*n* = 45)

Age, years	
Median (range)	66 (36–86)
Sex, *n* (%)	
Female	18 (40.0)
Male	27 (60.0)
Initial resectability	
Resectable	18 (40.0)
Borderline resectable	19 (42.2)
Unresectable	8 (17.8)
Histopathological diagnosis, *n* (%)	
Tubular adenocarcinoma	
Moredatery differentiated	35 (77.8)
Poorly differentiated	4 (8.9)
Undetermined	5 (11.1)
Adenosquamous Carcinoma	1 (2.2)
Stage (UICC, 8th ed)	
I	3 (6.7)
IIA	13 (28.9)
IIB	29 (64.4)
Recurrence, *n* (%)	
All recurence	25 (55.6)
Local recurrence	16 (35.6)
Peritoneal recurrence	8 (17.8)
Distant metastatic recurrence	14 (31.1)

UICC; Union Internationale Contre le Cancer

### Surgical margin sample collection procedures

After removing the surgical specimen by curative surgeries, float-on contamination of cells on the specimen's surface was removed by running water. Then, margin imprinting samples were collected by pressing 3 × 3 cm Hybond-C Extra nitrocellulose membranes (GE Healthcare, Little Chalfont, UK) directly on the specimen for 10 s following the previous publication [12, 13]. The membranes were placed into a coded 50 ml tube with 3 ml 1% SDS-PK solution. Three repeat membrane samples were taken from each facet of the specimen. Thereafter, matched tumor tissue and normal pancreatic ductal tissue were collected and placed in a coded 50 ml tube with 3 ml 1% SDS-PK solution.

### DNA extraction and bisulfite treatment

Collected tissues and margin imprinting samples were prepared by four rounds of proteinases K exposure during two overnight periods. These chemically digested samples were applied to dry bead tubes of UltraClean Tissue and Cells DNA isolation Kit (MO BIO Laboratories, Carlsbad, CA) for DNA extraction. One micro gram of DNA samples was subjected to bisulfite treatment by BisulFlash DNA Modification Kit (Epigentek, Farmingdale, NY).

### Quantitative methylation-specific PCR

*CD1D*, *KCNK12* and *PAX5* were selected as candidate excellent methylation markers for pancreatic adenocarcinomas extracted from the published previous papers [15, 16]. *ACTB* was measured for normalization. Primers and probes of target genes were set at CpG rich region, while those of *ACTB* were set at no CpG region (Additional file 2: Table S1). The bisulfite-modified DNA was used as a template for fluorescence-based QMSP. It was performed in triplicate using StepOnePlus (Thermo Fisher Scientific, Waltham, MA). Thermal cycling was initiated with denaturation at 95 °C for 10 min, followed by 40 cycles of 95 °C for 15 s and 60 °C for 1 min. Each plate included patient DNA samples, serially diluted positive standards of Bisulfite Converted Universal Methylated Human DNA Standards (Zymo Research, Irvine, CA) for constructing the standard curve, and multiple water blanks as no-template controls. Mean values of triplicate samples were used for analyses. The methylation ratio (QMSP value) is defined as the ratio of the fluorescence emission intensity values for the target gene-specific PCR products to those of the *ACTB* (reference gene) and then multiplied by 100 for easy tabulation. QMSP positive was defined as ≥ 1.0 following the previous work [13].

### Statistical analysis

Continuous variables were analyzed by the Mann–Whitney *U* test as a non-parametric test and Student's *t* test (2-tailed) as a parametric test. Categorical variables were analyzed by Fisher's exact test. RFS was defined as the time from surgery to first documentation of disease recurrence. OS was defined as the time from surgery to the date of death from any cause. RFS and OS were analyzed by the Kaplan–Meier method and compared using the log-rank test. Gray's test was used to evaluate the cause-specific cumulative incidence. Associations of gene methylation and other histopathological factors with RFS were evaluated by the Cox proportional hazards model with hazard ratios (HRs) and 95% confidence intervals (95%CIs). Associations with *p* < 0.05 in univariate analyses were further evaluated in multivariate regression analyses. All tests were 2-sided and considered statistically significant and clinically promising for values of *p* < 0.05. Statistical analyses were carried out with R version 3.3.2. (https://www.r-project.org/).

## Supplementary Information

**Additional file 1**. **Figure S1** Representative assays of molecular surgical margin analysis were shown. All of the margin samples had an amplification curve on the *ACTB* assay as a reference gene. While, some of them had the curve on some methylation signals, defined as MSM positive (a). Others had non of methylation signals, which was defined as MSM negative (b). **Figure S2** Recurrence-free survival rate (a), Overall survival rate (b) and recurrence rate (c-e) in patients with pathologically surgical margin negative (n=27) were analyzed.

**Additional file 2**. QMSP primers and probes of target genes and ACTB gene were listed.

## Data Availability

All data generated or analysed during this study are included in this article.

## Abbreviations

MSM: Molecular surgical margin analysis
NAT: Neoadjuvant therapy
RFS: Recurrence-free survival
OS: Overall survival
