# Supplementary material for: Impact of molecular surgical margin analysis on the prediction of pancreatic cancer recurrences after pancreaticoduodenectomy
Source: Clin Epigenetics. 2021 Sep 16;13:172. doi: 10.1186/s13148-021-01165-8 (PMC8444591; doi:10.1186/s13148-021-01165-8)

Figure S1. Representative results of molecular surgical margin analysis

a) Representative molecular surgical margin positive case

ACTB

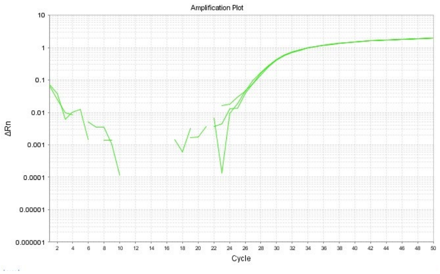

CD1D methylation

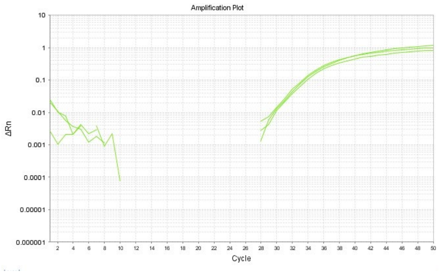

KCNK12 methylation

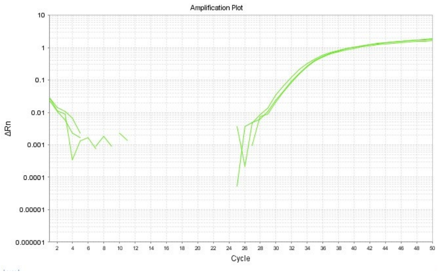

PAX5 methylation

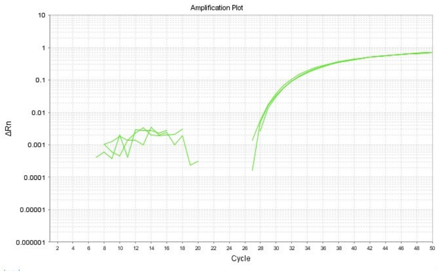

b) Representative molecular surgical margin negative case

ACTB

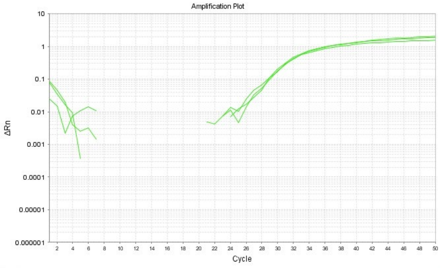

CD1D methylation

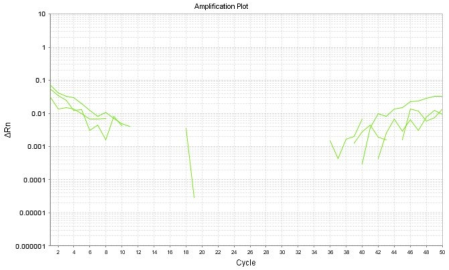

KCNK12 methylation

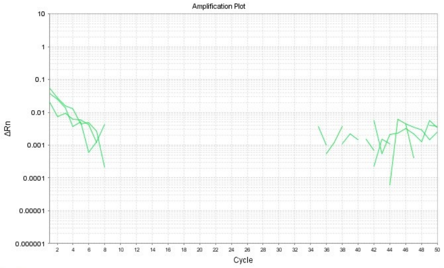

PAX5 methylation

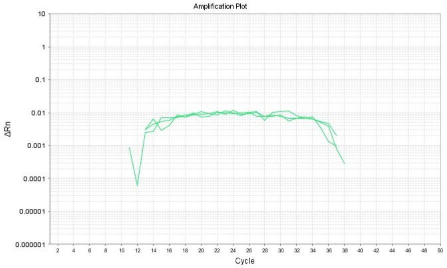

Figure S2. Survival and recurrence rate in patients with pathologically surgical margin negative

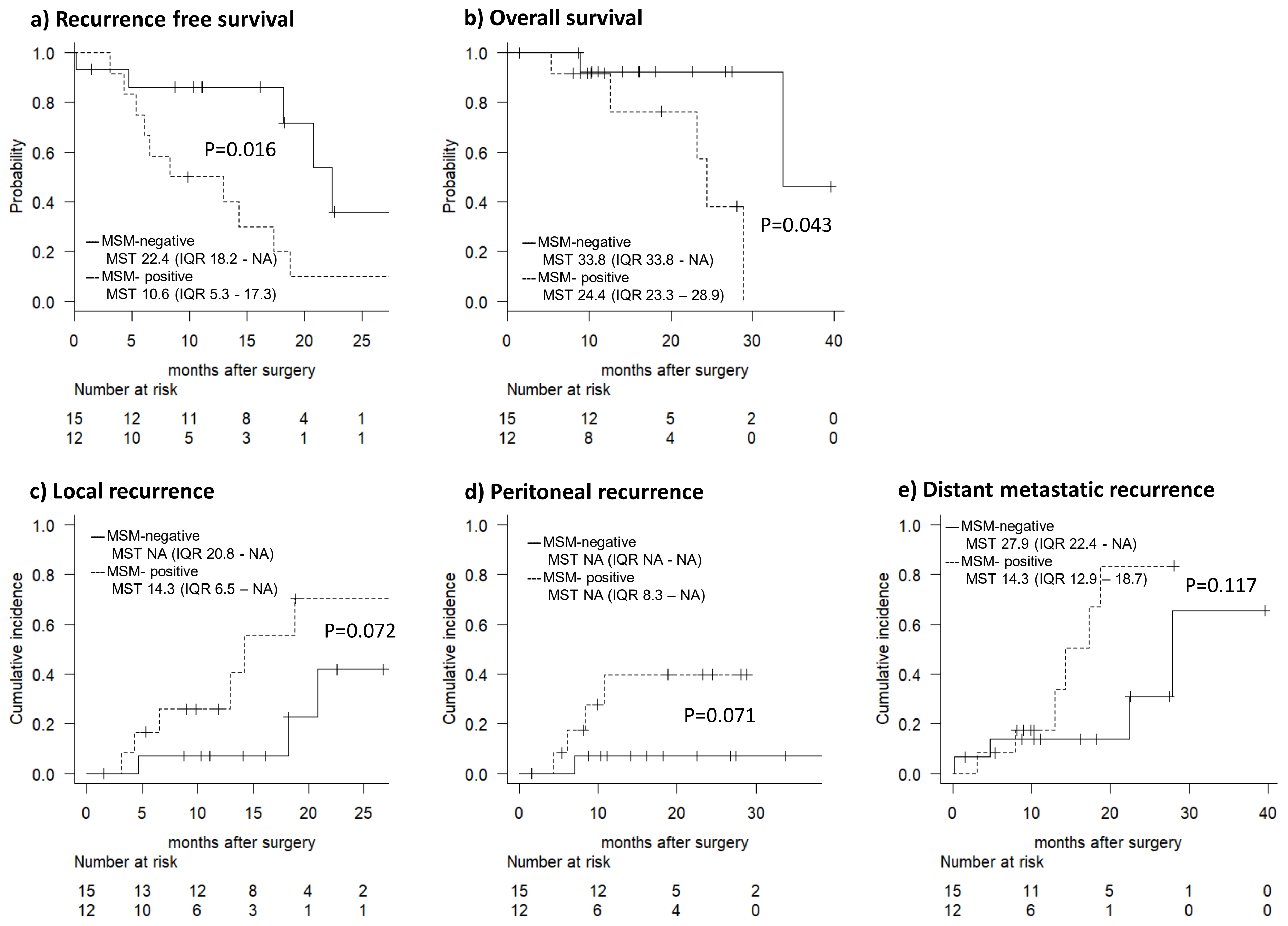

Supplement: Supplementary file 1 — Additional file 1. Figure S1 Representative assays of molecular surgical margin analysis were shown. All of the margin samples had an amplification curve on the ACTB assay as a reference gene. While, some of them had the curve on some methylation signals, defined as MSM positive (a). Others had non of methylation signals, which was defined as MSM negative (b). Figure S2 Recurrence-free survival rate (a), Overall survival rate (b) and recurrence rate (c-e) in patients with pathologically surgical margin negative (n=27) were analyzed. [file 13148_2021_1165_MOESM1_ESM.pdf]
