# Supplementary material for: Impact of molecular surgical margin analysis on the prediction of pancreatic cancer recurrences after pancreaticoduodenectomy
Source: Clin Epigenetics. 2021 Sep 16;13:172. doi: 10.1186/s13148-021-01165-8 (PMC8444591; doi:10.1186/s13148-021-01165-8)
Supplement: Supplementary file 2 — Additional file 2. QMSP primers and probes of target genes and ACTB gene were listed. [file 13148_2021_1165_MOESM2_ESM.pdf]

Supplementary Table S1. List of primers and probes used for QMSP

Gene	Forward 5'-3' (primer)	Probe (FAM-5', 3'-BHQ)	Reverse 5'-3' (primer)	Temperature	Amplicon
CD1D	TCG GTA GAA GTA GTA AAT CGT C	AAG TTT AGC GAG GAG GGT TGT CGG	CTC TAA CCT ACG CAC TCT TC	60 °C	89bp
KCNK12	TCG GAT TCG GTT CGG TTT C	ACG AAC AAT CCT ACT CCC GCC C	CGA CGC CTC CTA AAT ACA AAC TA	60 °C	59bp
PAX5	GCG TAA GAG AGA CGA AGG TAA G	AGA GGT TCG CGT AGT TTC GTC GG	ATA TTC GCG AAC ACC TCT ACT AC	60 °C	112bp
ACTB	TGG TGA TGG AGG AGG TTT AGT AAG T	ACC ACC ACC CAA CAC ACA ATA ACA AAC ACA	AAC CAA TAA AAC CTA CTC CTC CCT TAA	60 °C	133bp
